# Supplementary material for: Identification of photoperiod-induced specific miRNAs in the adrenal glands of Sunite sheep (Ovis aries)
Source: Front Vet Sci. 2022 Jul 22;9:888207. doi: 10.3389/fvets.2022.888207 (PMC9354845; doi:10.3389/fvets.2022.888207)
Supplement: Supplementary file 1 [file Data_Sheet_1.ZIP › Supplementary file/miRDeep2 file.zip!/miRDeep2 file/Know_Structure/LP42A3/oar-mir-26a.pdf]

miRBase precursor	: oar-mir-26a
Total read count	: 1341903
oar-miR-26a read count	: 1341768
remaining reads	: 135

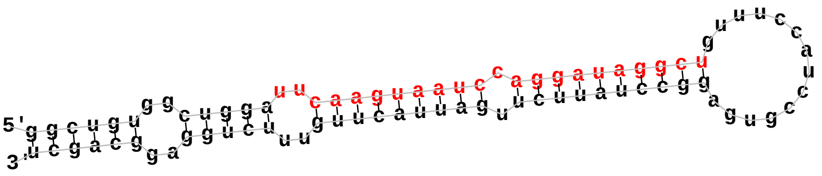

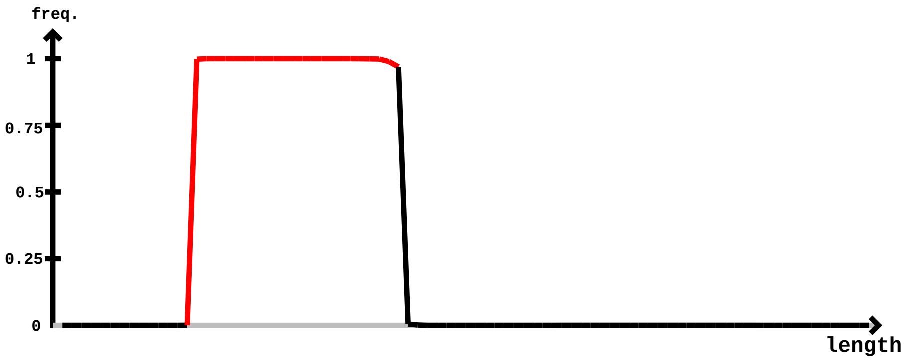


```
                oar-miR-26a
5'- ggcuguggcuggauucaaguaauccaggauaggcuguuuccauccgugaggccuauucuugauuacuuguuucuggaggcagcu   -3'    exp
    ((((((..(((((..(((((((((.((((((((((..............)))))))))).)))))))))..)))))..))))))    reads      mm         sample
    ............auucaaguaauccaggauagg...................................................    8          0          seq
    ............auucaaguaauccaggauaggc..................................................    9          0          seq
    ............auucaaguaauccaggauaggcu.................................................    236        0          seq
    ............Guucaaguaauccaggauaggcu.................................................    29         1          seq
    .............uucaaguaauccaggaA......................................................    10         1          seq
    .............uAcaaguaauccaggau......................................................    3          1          seq
    .............uucaaguaauccaggau......................................................    397        0          seq
    .............uucaaguaauccaggCu......................................................    2          1          seq
    .............uuAaaguaauccaggau......................................................    1          1          seq
    .............uucaaguaauccaggaG......................................................    5          1          seq
    .............uucaGguaauccaggau......................................................    3          1          seq
    .............uucaUguaauccaggau......................................................    2          1          seq
    .............uucaaAuaauccaggaua.....................................................    1          1          seq
    .............uucaaguaauccaggaua.....................................................    589        0          seq
    .............uucaGguaauccaggaua.....................................................    4          1          seq
    .............uucaaguaauccaggauag....................................................    666        0          seq
    .............uucaaguaauccaggauaC....................................................    6          1          seq
    .............uucaaguaauccaggauagg...................................................    12728      0          seq
    .............uucaaguaauccaggauaggc..................................................    26823      0          seq
    .............uucaaguaauccaggauaggcu.................................................    1291269    0          seq
    .............uucaaguaauccaggauaggcug................................................    4834       0          seq
    .............uucaaguaauccaggauaggcugu...............................................    1587       0          seq
    .............uucaaguaauccaggauaggcuguG..............................................    6          1          seq
    .............uucaaguaauccaggauaggcuguu..............................................    28         0          seq
    .............uucaaguaauccaggauaggcuguuu.............................................    2          0          seq
    .............uucaaguaauccaggauaggcuguuuc............................................    1          0          seq
    ..............ucaaguaauccaggaua.....................................................    3          0          seq
    ..............ucaaguaauccaggauag....................................................    1          0          seq
    ..............ucaaguaauccaggauagg...................................................    19         0          seq
    ..............ucaaguaauccaggauaggc..................................................    47         0          seq
    ..............ucaaguaauccaggauaggcu.................................................    2302       0          seq
    ..............ucaaguaauccaggauaggcug................................................    24         0          seq
    ..............ucaaguaauccaggauaggcugu...............................................    16         0          seq
    ...............caaguaauccaggauagg...................................................    2          0          seq
    ...............caaguaauccaggauaggc..................................................    1          0          seq
```

oar-miR-26a

```
ggcuguggcuggauucaaguaauccaggauaggcuguuuccauccgugaggccuauucuugauuacuuguuucuggaggcagcu
...............caaguaauccaggauaggcu.................................................    85    0    seq
...............caaguaauccaggauaggcug................................................    1     0    seq
...............caaguaauccaggauaggcugu...............................................    1     0    seq
................aaguaauccaggauagg...................................................    1     0    seq
................aaguaauccaggauaggcu.................................................    14    0    seq
................aaguaauccaggauaggcuguu..............................................    1     0    seq
.................aguaauccaggauaggcu.................................................    1     0    seq
.............................................gugaggccuauucuugauuacu.................    1     0    seq
................................................aggccuauucuugauuacuugu..............    2     0    seq
................................................aggccuauucuugauuacuuguu.............    4     0    seq
................................................aggccuauucuugauuacuuguuu............    1     0    seq
...................................................ccuauucuugauuacuuguu.............    5     0    seq
...................................................ccuauucuugauuacuuguuu............    66    0    seq
...................................................ccuauucuugauuacuuguuuc...........    50    0    seq
...................................................ccuauucuugauuacuuguuucu..........    5     0    seq
....................................................cuauucuugauuacuuguuuc...........    1     0    seq
```
